# Proteomic and Microscopic Strategies towards the Analysis of the Cytoskeletal Networks in Major Neuropsychiatric Disorders

**DOI:** 10.3390/ijms17040581

**Published:** 2016-04-20

**Authors:** Joëlle V. F. Coumans, Suresh K. A. Palanisamy, Jim McFarlane, Pierre D. J. Moens

**Affiliations:** 1 School of Rural Medicine, University of New England, Armidale, NSW 2351, Australia; 2 Center for Bioactive Discovery in Health and Aging, School of Science and Technology, University of New England, Armidale, NSW 2351, Australia; sureshkumarap.armidale@gmail.com (S.K.A.P.); jmcfarla@une.edu.au (J.M.); pmoens@une.edu.au (P.D.J.M.)

**Keywords:** neuropsychiatric disorders, cytoskeleton, cytoskeleton associated proteins, proteomics methodologies, cytoskeleton protein enrichment, live cell imaging, super-resolution microscopy

## Abstract

Mental health disorders have become worldwide health priorities. It is estimated that in the next 20 years they will account for a 16 trillion United State dollars (US$) loss. Up to now, the underlying pathophysiology of psychiatric disorders remains elusive. Altered cytoskeleton proteins expression that may influence the assembly, organization and maintenance of cytoskeletal integrity has been reported in major depressive disorders, schizophrenia and to some extent bipolar disorders. The use of quantitative proteomics, dynamic microscopy and super-resolution microscopy to investigate disease-specific protein signatures holds great promise to improve our understanding of these disorders. In this review, we present the currently available quantitative proteomic approaches use in neurology, gel-based, stable isotope-labelling and label-free methodologies and evaluate their strengths and limitations. We also reported on enrichment/subfractionation methods that target the cytoskeleton associated proteins and discuss the need of alternative methods for further characterization of the neurocytoskeletal proteome. Finally, we present live cell imaging approaches and emerging dynamic microscopy technology that will provide the tools necessary to investigate protein interactions and their dynamics in the whole cells. While these areas of research are still in their infancy, they offer huge potential towards the understanding of the neuronal network stability and its modification across neuropsychiatric disorders.

## 1. Introduction

Historically, and up to recently, mental illnesses were not global health priorities when compared with infectious/contagious diseases and conditions such as cancers, cardiovascular disease, diabetes and chronic lung illnesses. The 2010 Global Burden of Disease (GBD) study has revealed that neuropsychiatric conditions were significantly responsible for the total disease burden in the world as measured in years lived with disability (YLD); depression being the most disabling disorder worldwide. This was nicely summarized by the review of Whiteford *et al.* [1], which emphasized that mental health and substance use were the fifth leading disorder category of global disability-adjusted life-years (DALYs) accounting for 183.9 million DALYs and equivalent to 7.4% of total disease burden in 2010. Recently, the report by the World Economic Forum and the Harvard School of Public Health estimated that in the next 20 years mental health conditions alone will account for the loss of US$16 trillion, equivalent to 25% of global Gross domestic product (GDP) in 2010 [2]. Despite these critical data, up to now, molecular mechanisms underpinning neuropsychiatric disorders remain largely misunderstood and pursuing novel strategies for the treatment and even prevention of mental illness is of foremost importance.

The organization of the nervous system relies on the establishment of neuronal polarity, which is essential for the unidirectional signal flow from dendrites to axons. This involves several discrete steps: (1) migration of the newborn neurons into their proper locations; (2) establishment of a polarized neuron by developing a single axon; (3) elongation of the remaining neurites into dendrites; and (4) formation of synapses with appropriate partners to establish a neuronal network [3]. All of these processes rely on the specific and coordinated dynamics and organization of the cytoskeleton. A desire to understand the mechanisms underlying this coordinated regulation is shared among biologists and biophysicists. Because of their open-ended, hypothesis-free nature, proteomic approaches have provided useful information into the molecular alterations occurring in neuropsychiatric disorders and suggested that cytoskeletal integrity of neurones might be disrupted to at least some extent, as reviewed below. The brain proteome is highly complex. Indeed, it is estimated that about 20,000 genes are expressed and more than 300 potential posttranslational modifications, each of which can affect protein functioning [4]. This complexity with its million-fold dynamic range, and, at the analytical level, the diversity of physical properties that a protein can have, have delayed our ability to obtain a comprehensive unbiased discovery of protein systems and their relation with cell behaviours, highlighting the need of new approaches.

Therefore, beside reporting on the contribution of proteomics to quantitative discovery research in psychiatric disorders, we discuss the need of alternative methods for further characterization of the neurocytoskeletal proteome and present live cell dynamic microscopy technologies that will provide the tools necessary to investigate protein interactions and their dynamics in the whole cells.

## 2. The Cytoskeletal Systems

The cytoskeleton is an adaptive and dynamic cellular network of protein polymers composed of three distinct but highly intertwined filamentous structures: microfilaments (MFs), intermediate filaments (IFs) and microtubules (MTs). They differ in term of structure, turnover dynamics, spatial organisation and function. MFs and MTs both display a polarity in subunit orientation. MFs (7–10 nm in width) consist of two actin protofilaments twisted around each other in a right-handed helix and MTs (14 nm in width) derive from the assembly α- and β- tubulin subunits in a head-to-tail fashion. Both of these structures exhibit an out-of-equilibrium polymerization process. At steady-state phase, MFs polymerization of actin monomers (G-actin) into filaments (F-actin) occurs according to a process referred to as “treadmilling” where free-floating G-actin continually associates at the fast-growing barbed or “plus end” and dissociates from the slower-growing pointed end, or the “minus end” [5]. Polarity in MTs results from a difference in tubulins subunits at both ends. The α-tubulin subunits are always bound to guanosine triphosphate, conferring to this subunit a conformation suitable for MT polymerization, whereas the β-tubulin subunit can be bound either to guanosine triphosphate or guanosine diphosphate, which is favourable for MT polymerization or de-polymerization, respectively. Therefore, cycles of growth and shrinkage occurs more rapidly at the β-tubulin exposed end or “plus end” compare to the α-tubulin exposed end or “minus end” where exchange is relatively slow [6]. Additionally, MFs and MTs dynamics are regulated by interactions with others proteins.

Actin-formed structures are modified by various kinds of actin-binding proteins (ABPs). For example, thymosin-β4, actin depolymerizing factor (ADF)/cofilin and profilin depolymerize filaments or bind G-actin subunits increasing MFs instability [7]. Arp2/3 complex nucleate MFs to form a branching network that acts as the supporting architecture in neurons [8,9]. MTs dynamic is directly or indirectly controlled by MT-associated proteins (MAPs) and MT-interacting proteins, which act either by stabilizing/destabilizing MT, promoting their assembly, depolymerisation or fragmentation, or controlling tubulin availability. Moreover, interactions between MTs and MAPs are influenced by diverse posttranslational modifications (PTMs) [6]. In contrast, IFs (10 nm diameter), in neurons also referred to as neurofilaments, are apolar structure consisting of a highly conserved central α-helical rod domain and variable N-terminal head and C-terminal tail domains with remodelling regulated by protein phosphorylation [10,11].

Cell morphology and movement, phagocytosis, endocytosis, cell-to-cell and cell-to-matrix attachments are functions generally attributed to MFs which are found principally in presynaptic terminals and growth cones and are abundant in dendritic spines where they are considered to be a determining factor for dendritic spine morphology and synaptic function [9,12]. IFs are generally considered to confer cell stiffness and strength and being responsible to maintain cellular shape. Finally, MTs are mainly involved in intracellular transport of organelles and the formation of the mitotic spindle and therefore considered essential regulators of neuronal morphogenesis.

## 3. The Cytoskeleton in Neuropsychiatric Disorders

**Figure 1:**
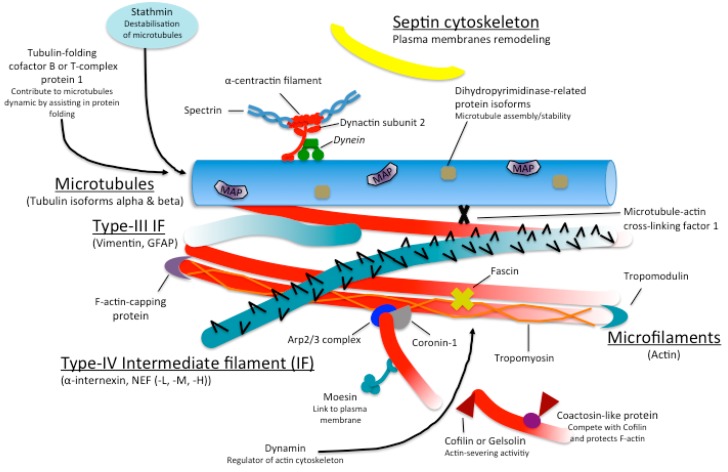
Illustrate the function/association of the differentially expressed cytoskeletal proteins in schizophrenic patients. This diagram has been updated from [18].

Dysregulation of the cytoskeleton and its dynamics has been associated with a number of psychiatric disorders. In schizophrenia (SCZ), altered cognitive and affective functions, as well as reduced dendritic spine density and diminished synaptic connectivity are present in the prefrontal cortex and in the limbic system. Structures in the limbic system play key roles in affective and cognitive functions; therefore altered cytoskeletal organization and a disturbance of the neuronal polarity may be critical in the unusual behaviour observed in SCZ patients [13]. Recent studies support the idea that aberrant cytoskeletal organization, which underlies the pathologic lesions of SCZ, may, in part, be due to altered microtubule-associated proteins (MAPs). Indeed, it has been observed that MAP-2 and -3 are abnormally expressed and that there is altered phosphorylation of MAP1B [14]. Moreover, it has been proposed that depletion of MAP6 can cause impairment of cognitive function [15]. Proteomic analysis of different portion of SCZ brain (see Table 1 and Figure 1) revealed alterations of tubulin subunits (TUBB), the glial fibrillary acidic protein (GFAP), which is the major IF protein of mature astrocytes, the dopamine receptor interacting protein Neurofilament, Medium Polypeptide (NEFM) and the N-methyl-D-aspartate receptor (NMDA) receptors associated protein Neurofilament, Light Polypeptide (NEFL) as well as dynamin 1 (DNM1), a protein involved in receptor-mediated endocytosis, that could be responsible for dysfunctions of both dopaminergic and glutamatergic receptors observed in SCZ and to the effects of antipsychotic drugs [16]. Deficiency in coronin-1 (CORO1), interestingly also downregulated in SCZ (Table 1), has been shown to induce loss of excitatory synapses and severe neurobehavioral disabilities, including reduced anxiety, social deficits, increased aggression, and learning defects [17]. Dihydropyrimidinase Like Protein (DPYSL) 2 and DPYSL3 were differentially expressed in both grey and white matter of SCZ patients. DPYSL2 is known to play key roles in axonal growth and branching during neuronal development [18] while DPYSL3 promote cell migration by regulating F-actin bundling [19]. DPYSL2 phosphorylation influences its binding to tubulin. Non-phosphorylated DPYSL2 favors microtubule formation, whereas its phosphorylation by Rho kinase suppresses its binding to tubulin [20]. In addition, DPYSL2 regulates the stability of actin filaments through its interaction with the Sra-1/ /WASP family verprolin-homologous protein 1 (WAVE1) complex [21]. Moreover, it has been shown recently that DPYSL2 and DPYSL3 work co-ordinately through their interaction with microtubules and actin to regulate growth cone development and axon elongation, two critical steps in the establishment of perfect neuronal circuit [22]. Interestingly, alterations in DPYSL2 and DPYSL3 expression levels were the most spectacular consequences observed in rat cerebral cortex proteins after chronic administration of two antipsychotic drugs, clozapine and risperidone [23]. In 2006, Beasley *et al.* [24] reported that the protein levels of DPYSL2 correlated significantly with antipsychotic exposure and, in 2013, Pickering *et al.* [25] reported that chronic treatment of rat with phencyclidine, a drug that mimic schizophrenia symptoms, downregulates DPYSL2 and upregulates DPYSL3 levels in the medial prefrontal cortex. Despite these encouraging observations, we must also look beyond proteomic-based methods in order to understand the extent to which these proteins are associated with schizophrenia and provide further confirmation.

Major depressive disorder (MDD) is another serious psychiatric condition and although some aspects of MDD have been identified, such as hypothalamic-pituitary-adrenal axis dysfunction [43] effects on memory [44] and volume reduction of certain brain regions such as hippocampus [45] and prefrontal cortex [46], the underlying pathophysiology of this disorder still needs elucidation. MDD is typically marked by repeated episodes of low mood and often accompanied by other psychiatric disorders such as anxiety disorders, obsessive-compulsive disorder, dementia, and SCZ. Morphometric alterations in the limbic system as well as volume loss in the hippocampus of depressed individuals suggest a possible involvement of structural neuronal plasticity [47]. Chronic stress is one of the important risk factors for depression. Chronic stress animal models show alteration in tubulin and actin as well as in their associated proteins as reviewed recently by Wong *et al.* [48]. Only few proteomic studies on two brain regions, the frontal cortex (FC) and the anterior cingulated cortex (ACC), from depressed patients have been published [24,26,49,50]. Two of these reported altered expression of tubulin isoforms and MAP (Table 2). Additionally, Martin-de-Souza *et al.* [50] reported altered expression of phosphopeptides belonging to these proteins, suggesting the importance of post-translational modification (PTM) in the cause of cytoskeletal dysfunction/disturbance associated with depression. Common among three of these studies is the altered expression of DPYSL2 (Table 2). DPYSL2 is known to be involved in axon guidance regulation, vesicle trafficking and synaptic function, and is modulated by antidepressants and neuroactive molecules such as the brain derived neurotrophic factor (BDNF) [51]. BDNF, which is known to blocks phosphorylation of DPYSL2 via the PI3-kinase/AKt/GSK3-β pathway [52], is decreased in the brain and serum of patient with depression [53,54]. Phosphorylated DPYSL2 has a decreased affinity for tubulin heterodimers that leads to a diminution in microtubule growth and, as a consequence axon retraction [51], additionally knockdown of DPYSL2 with siRNA inhibited BDNF-induced axon outgrowth and branching [52]. Taken together, these observations suggest that DPYSL2 might play a role in MDD physiopathology. However, contradicting results have been reported in these works, with 2 studies reporting an increase in DPYSL2 expression levels and one reporting a decreased expression. In addition, the phosphoproteomic study of Martin-de-Souza *et al.* [50] reported a decrease in expression of 2 phosphopeptides of DPYSL2. Therefore, further work is needed to clarify the exact role of DPSLY2 in MDD. Profilin (PFN1 and PFN2) is another protein with altered expression in MDD [49]. Recently, Focking *et al.* [55] reported an upregulation of PFN1 in their study of the mice hippocampus following prenatal stress, a condition known to increase the risk of the development of depression and schizophrenia in adult offspring. PFN1 being a promoter of actin polymerization, these data suggest the occurrence of MFs remodelling in MDD pathology.

Bipolar disorder (BPD) is a mental illness in which depression and mania typically alternate, and both phases can present with psychotic features. The symptomatology of BPD, therefore, resembles MDD and SCZ. It is therefore logic to think that BPD can also be associated to some extend with cytoskeletal modifications of the brain. Morphometric studies reported a decrease of neuronal and glial density in association with glial hypertrophy in the white matter of BPD patients [56,57]. Recently, a transcriptome sequencing study by Zhao *et al.* [58] provided evidence of interconnected pathway networks common to both SCZ and BPD. Based on differentially and concordantly expressed genes, one of the pathways identified is the regulation of the actin cytoskeleton. The few proteomic studies that reported on the differential expression of proteins in BPD (Table 3) reveal differential expression of cytoskeletal associated-proteins with several of them being common across SCZ, MDD and BDP (Table 1, Table 2 and Table 3), suggesting commonality but also difference between these diseases.

Therefore, an understanding of the molecular mechanisms that underlie cytoskeletal and cytoskeletal associated protein expressions as well as modifications in their PTM is critical towards the understanding of the development/control/maintenance of the neuronal network stability which is modified across most neuropsychiatric disorders. Because these disorders have a complex aetiology, homeostatic modifications of several proteins need to be suspected rather than a single one to explain the behavioural phenotypical changes. Quantitative, high-throughput, non-hypothesis-driven experimental approaches are therefore required. Particularly, quantification of proteins as it is increasingly appreciated that mRNA and protein changes correlate poorly [59,60,61], accentuating the importance of protein expression analysis to fully characterize disease pathways. Proteomics technologies, as well as advances made in these technologies, are well suited to take on this challenge and have already highlighted important candidates towards a molecular understanding of these diseases as reviewed above. Coupled with proteomics investigations, understanding the dynamics and interplay between the identified proteins in live cells is the next step towards the generation of efficient treatments. Recent advances in dynamic microscopy and super-resolution microscopy are providing new tools that will bring the understanding of these processes within our reach.

## 4. Proteomic Methodologies Used and Emerging

The term proteomics was coined to make analogy with “genomics”. It was originally defined as “the study of the total set of expressed proteins by a cell, tissue or organism at a given time under a determined condition” [62]. Since then, other aspects have been included such as the characterization and identification of post-translational modifications, the understanding of protein–protein interactions, organization, and networks and more.

The relative quantification of proteins between samples of interest is probably the most popular tool in the proteomics field. To date, there are two main quantitative methods that are viewed as sufficiently high throughput and reproducible: the traditional, two-dimensional gel electrophoresis (2-DE) method and the more recently developed, liquid chromatography/mass spectrometry (LC/MS)-based approaches. These approaches have advantages and disadvantages, and their combination seems to be the best strategy to adopt, as they appear to be complementary [63].

2-DE, which has provided the basis of most proteomic discovery in SCZ, MDD and BP studies (Table 4), has been employed for protein separation since 1975 [64], and further optimized (for review: [65,66]).

The principle of this method is to separate the proteins by two of their physicochemical characteristics: in the first dimension according to their charge or isoelectric point (pI), generally in a gel with an immobilized pH gradient and in the second dimension according to their molecular weight (MW) using SDS-polyacrylamide gel electrophoresis (SDS-PAGE). Visualization of the proteins is generally achieved by Coomassie Blue or silver staining or as more recently developed by labelling of the proteins with fluorescent dyes prior to electrophoresis, also known as 2D fluorescence difference gel electrophoresis (2D-DIGE) [67]. 2D-DIGE, which offer a more precise and more sensitive quantification (approximately tenfold less sample compare to base stain 2-DE), rely on the labelling with mass- and charge-matched cyanine-derived fluorophores (termed Cy2, Cy3 and Cy5), which bind covalently with free amines on proteins. The labelled proteins are then combined into a single sample that is separated by 2-DE. This process therefore minimizes technical variations as both samples are subjected to identical running conditions. In addition, the availability of a third dye allows the incorporation of an internal standard for normalization purposes and for multi-gel comparisons. After 2-DE, the gels are digitalized and compared with the help of specialized computational software. Intensity and volume of individual protein spot is determined and cross-gel comparison is performed with the aim of identifying differentially expressed protein spots. Spots of interest are then excised from the gels, digested, and identified by mass spectrometry (MS.) Overall 2-DE gel-based methods are robust and reproducible but somewhat labour-intensive and drawbacks, such as the potential overlap of proteins in a single spot, the difficult resolution of low abundant, hydrophobic, very acidic, very basic, very small, and very large proteins as well as sample solubility issues has lead to the development of shotgun-MS approaches. These approaches, while being less reproducible, are capable of analysing more complex protein samples with a superior coverage. This shift of the proteomics field towards MS is due to the ability to identify proteins by measuring the molecular mass-to-charge (*m*/*z*) ratio of ions (*i.e.*, molecules or peptides that have been electrically charged) [68]. In LC/MS approaches, proteins are first digested; usually using trypsin, separated by reverse-phase chromatography and analysed by MS/MS. Identification of the proteins is achieved by automated assignment of acquired MS/MS spectra to predicted spectra available in protein sequences databases [69]. One of the drawbacks of peptide-based analysis is sample complexity. A tryptic digest of an average protein will yield 20 or more peptides. Taking into account that some of these peptides might carry post-translational modifications, a single gene product might present with multiple masses. Therefore, obtaining a comprehensive analysis of a proteome is highly dependent of the ability of the mass spectrometer to deal with this heterogeneity within a reasonable time frame. Hence, the fact that tissue samples are generally a mixture of different cell types makes the discovery of biomarkers or arbitrators of diseases highly challenging, as the most abundant proteins are not necessarily involved. To alleviate some of these challenges, *in vitro* stable isotope labelling methods, such as isobaric tags for relative and absolute concentration (iTRAQ) [70], isotope-coded affinity tags (ICAT) [71] and isotope-coded protein labelling (ICPL) [72] or *in vivo* approaches, such as stable isotope labelling with amino acids (Lys and Arg) in cell culture (SILAC) [73] or more recently developed the stable isotope labelling in mammals (SILAM) [74] have been developed and used to quantify proteins in MS comparative analysis. iTRAQ is often the method of choice for *in vitro* labelling as it offers the unique opportunity to compare up to eight samples in the same experiment. Although these methods are well established in other research fields, only few papers reported their use in neuropsychiatric proteomic research (Table 4). Emerging in the neuropsychiatric proteomic field is the use of MS experiments that do not require proteins or peptides to be labelled [42,49]. The appeal of this approach rely on the fact that not only labelling is not required but also there are no limits regarding the number of samples that can be compared, which is an advantage as it provides greater statistical power. The so-called label-free proteomics rely on the theoretical assumption that the chromatographic peak area of a peptide correlates with its concentration [75]. To date two different quantification methods have been used: (i) area under the curve (AUC) or signal intensity measurement based on precursor ion spectra; and (ii) spectral counting, which is based on counting the number of peptides assigned to a protein in an MS/MS experiment [76].

Until now, most proteome studies analysing major neuropsychiatric disorders have used a profiling approach with the aim to gain a comprehension of these diseases as well as to identify diagnostic/prognostic biomarkers. Taken together, their results have increased our understanding of the affected molecular pathways underpinning these diseases and have confirmed that cytoskeleton deregulation occurs in, SCZ, MDD and BPD. A major drawback of these studies is their lack of analytical depth, which is inherent to the complexity of the brain proteome, as well as the scarcity of adequate validation and follow-up analysis. This has resulted in an incomplete view of the specific modifications occurring in a disease and a partial understanding of modified pathways.

One possibility to alleviate these problems is to implement more targeted proteomics approaches. A way to tackle the problem is to improve sample preparation by enrichment/subfractionation methods targeting the cytoskeletal-associated proteins. Differential detergent fractionation (DDF), which relies on detergents to sequentially extract proteins, has been used to isolate cytoskeletal-associated and interacting proteins [77]. Other studies have shown that the cytoskeleton and its associated proteins could be selectively enriched using Dynal beads [78,79]. Tubulin and actin affinity chromatography have been used to enrich samples with their binding partners [80,81,82] and MT-associated proteome has been investigated by *in situ* taxol-enhanced tubulin polymerization and purification by centrifugation [83]. In our lab, we are currently adapting a method originally developed by Phung-Koskas *et al.* [84] that allowed the isolation of unpolymerized tubulin and unbound MAPs as well as the isolation of total MT and their associated MAPs. Taken together, enrichment procedures for characterization of the cytoskeletal proteome are available but methodological studies comparing the currently available procedures that establish which method provides the highest recovery with minimal contamination are lacking. Another way to target a particular set of proteins is to use antibodies and other affinity reagents that can efficiently bind to their target independently of the sample complexity. Of course one of the main limitation of these approaches is the availability of suitable reagent. The Human Protein Atlas project currently demonstrates the feasibility of this task, as we are reaching proteome-wide collections of antibodies [85] or aptamers [86]. As review by Solier *et al.* [87] reverse-phase protein arrays (RPPAs) have been successfully used for efficient protein quantification, characterization of signalling pathways as well as PTMs analysis. Because of their stability and specificity, which can be easily adjusted at low cost, aptamers are gaining interest towards the replacement of antibodies in proteins detection.

In term of results validation, targeted MS-quantitative analysis, such as selected reaction monitoring (SRM) or multiple reaction monitoring (MRM) has gained popularity overcoming the laborious Western blot and ELISA methods. The basic principle of SRM/MRM is to set up the mass spectrometer to specifically detect only representative and unique peptides of a protein of interest for quantification. Performed either through a triple quadrupole linear ion trap mass spectrometer or trough the more recent TripleTOF 5600 form AB Sciex, SRM/MRM analysis have the ability to provide reproducibility, specificity and better quantitative accuracy in a high throughput format. However, it is clear that accurate quantification is only achieved if the selected peptides are fully ionized and produce intense fragment peaks which in reality is unfortunately rarely achieved as the presence of other peptides, which are injected at the same time, reduces the ionization efficiency and that post-translational modifications can affect both ionization and fragmentation. Nerveless, SRM/MRM are very attractive methods because they can also be used to analyse PTMs thus enabling cellular functional status of the sample(s) of interest to be studied [88]. To our knowledge, only one study by Wesseling *et al.* [41] reported on the validation of 56 brain proteins previously implicated in SCZ, MDD and BP by SRM.

## 5. Microscopic Technologies Used and Emerging

The generation of fusion proteins with green fluorescent proteins and its variants have made possible to track and observe expressed proteins inside living cells. Time-lapse fluorescence imaging has been one of the most important approaches in neurobiological research with the use of 2-photon microscopy and confocal microscopy to study for instance the dynamics of the cytoskeleton in synaptic morphology [89]. The lateral resolution of these systems are around 250 nm for the 2-photon microscope and 200 nm for the confocal microscope while the axial resolution is larger by a factor of 5 for the 2 photon and by a factor of 3 for the confocal microscope. These result in observation volumes of ~0.153 and ~0.047 µm^3^, respectively. Such observation volumes can therefore contain hundreds of proteins. Multicolour imaging and colocalisation of labelled proteins merely indicate that the proteins of interest are present in these particular volumes but do not provide information regarding their interactions or detailed structures which would require sub diffraction resolutions. The study of protein interactions is often achieved using Förster resonance energy transfer (FRET) microscopy or cross correlation spectroscopy. The amount of FRET is mostly determined from the intensity of the fluorescence of a donor molecule for instance linked to the protein of interest in presence of an acceptor linked to the binding partner compared to the fluorescence of the donor alone (obtaining the signal of the donor alone in intensity measurements can be achieved by bleaching the acceptor molecules) or using lifetime measurements of the donor in similar conditions. In this case and since the measurements do not depend upon the concentration of the donor, the lifetime of the donor alone can be determined in cells which have not been labelled with acceptor molecules. However, lifetime images can be difficult to analyse since one has often to fit multi-exponential decays on every pixel of the images with a relatively low number of photons (~1000) collected in each pixel of the image and identifying decay times and amplitudes with molecular species and their relative abundances. Digman *et al.* [90] have developed a fit less approach to lifetime image analysis. They used the phasor approach [91,92,93] to graphically analyse the images. Using this approach, every pixel of the image is represented in a polar plot and the contribution of the auto fluorescence, the donor molecules in absence of acceptor and the amount of FRET (fluorescence of the donors in presence of acceptors) can be graphically evaluated. In correlation spectroscopy, FRET can be determined using two channels cross correlation analysis of single point FCS (fluctuation correlation spectroscopy) measurements [94]. This method however, has limited spatial resolutions since it only investigate the diffusion and molecular interaction of the molecules within the confocal volume in one point of the cell. Other approaches such as raster scan image correlation spectroscopy [95,96,97] and Number and Brightness analysis [95,96] have subsequently been developed to provide information such as diffusion coefficient, binding and number of molecules and aggregations states with spatial and temporal resolutions.

However, while all these methods have increased our understanding of the cytoskeleton and cytoskeleton associated proteins dynamics in living cells, these studies are currently limited to cell lines in cultures because of the lack of suitable fluorescent probes for tagging proteins of interest in live animal models of neuropsychiatric disorders. In addition, the resolution of these methods is limited by the diffraction of light forcing researchers to complement the live cell imaging strategy by electron microscopy (EM). In the last decade, several methods were developed to overcome this barrier and allow us explorations of neuronal structures including the cytoskeleton below the diffraction limit of light. Among these methods, we find PALM (photoactivated localization microscopy), STORM (stochastic optical reconstruction microscopy, STED (stimulated emission depletion), GSDIM (ground state depletion imaging microscopy) and their variations (for review see [98]). Some of these methods have already been successfully applied to the investigation of protein localization in neurons and synapses [99,100,101] and the application of multicolour imaging will allow the study of protein colocalization at the nanometre scale [102]. The joint improvement of resolution and colocalization permitted by these techniques demonstrates the emerging potential of superresolution microscopy to study the spatial organization of cytoskeleton-associated proteins in cells and neurons. A review of the applications of superresolution localization based microscopy in neurons has recently been published [103] highlighting the power but also the limitations of these techniques in neurosciences. One of these limitations is time resolution as these methods are not currently suitable to investigate fast dynamics due to the nature of the acquisition process. Single particle tracking provides high temporal and spatial resolution and are particularly successful at obtaining nanoscale dynamic information on component of the membrane. Application of single particle tracking has been growing in the field of neurobiology and has been used in this field to study for instance molecular surface dynamics in brain slices [104] or membrane protein dynamics in neurons [105]. Alcor and his colleagues [106] recently published a review of single particle tracking application in the study of membrane receptor dynamics. On the downside, single particle tracking rely on the recording and analysis of a large number of particle trajectories and often use large molecular markers such as gold particle or quantum dots which could limit heir access to confined compartments such as synaptic clefts [105]. Unfortunately, the use of organic dyes or fluorescent proteins has been limited in this technique due to their short fluorescence stability [106].

The image-Mean Square Displacement technique (iMSD) is a new method to study membrane protein dynamics, which is compatible with small organic dyes or fluorescent molecules. The method is based on the calculation of the Spatio-temporal Image Correlation function but introduces a quantity similar to the mean square displacement (MSD) used in single particle tracking while analysing the entire image and without separation of the particles. The iMSD *versus* time plot obtained is then used to reconstruct the protein “diffusion law” [107]. From this plot, one can directly identify and determine the number of molecules under observation and identify whether the labelled molecules are undergoing pure isotropic, restricted, corralled, or directed diffusion. Given that the size of the corrals can be determined with nanometre resolution, regions of confined barriers can be mapped in the cell. Because iMSD does not require the acquisition of a large number of trajectories, it can be applied to the entire cell under analysis. In addition, multi-colour cross correlation analysis is possible and therefore, dynamic interactions between proteins on the membrane can be investigated in live cells. Because iMSD is based on the use of total internal reflection microscopy (TIRF) it is currently limited to the investigation of membrane proteins or proteins that are closely associated to the membrane. The application of iMSD combined with light sheet fluorescence microscopy (LSFM) or selective plane illumination microscopy (SPIM) offering the capacity for fast 3D acquisition, low photobleaching and good penetration depth [108,109,110] would provide us with a tool capable of investigating the fast dynamics of cytoskeleton associated proteins and protein interactions in the whole cells with resolution not limited to the diffraction of light.

## 6. Conclusions

SCX, MDD and BPD have complex aetiology due to homeostatic modifications of many proteins of the cytoskeleton rather than a single one. Quantitative, high-throughput, non-hypothesis-driven experimental approaches are required in order to increase our understanding of these pathologies. The application of mass spectrometry-based methods such as iTRAQ and label-free as well as targeted SRM/MRM and antibodies/aptamers-arrays have great potential towards the profiling of MDD, SCZ and BPD. The use and the development of better enrichment procedures and/or alternative methods for further profiling of the cytoskeletal proteome will ultimately lead to the identification of molecular pathway involved in the regulation of the cytoskeleton. Once the proteins involved have been identified, the interaction and dynamics of the cytoskeleton associated proteins could further be investigated by live cell imaging and dynamic microscopy in order to understand the subtle interactions that are involved in its regulation. In conclusion, we have the tools necessary to investigate the mechanisms underlying the development/control/maintenance of the neuronal network stability and their modification across neuropsychiatric disorders. Future studies using new proteomic and microscopic methods have the potential to make a greater contribution to our understanding of these diseases at the cellular and molecular level.

## Figures and Tables

**Table 1:** Summary of the proteins altered expression from postmortem proteomic investigations of Schizophrenia. Gray matter (GM), white matter (WM), downregulated (↓), upregulated (↑).

			References																	
Protein Name	Gene Symbol	Uniprot acc. No.	[26]	[27]	[24]	[28]	[29]	[30]	[31]	[32]	[18]	[33]	[34]	[35]	[36]	[37]	[38]	[39,40]	[41]	[42]
Actin isoforms	ACTG1	P63261		↓GM and WM			↓		‒	‒		‒	‒				‒			
	ACTB	P02570					↓			↑		↓	‒				↓			
	ACTA1	P68133											↓							
α-centractin	ACTR1A	P61163					↑													
Actin-related protein 2/3 complex components	ACTR3	Q92747		‒		↑				↓										
	ARPC1A	P61158		↓ GM																
	ARPC1B	O15143																		
F-actin-capping protein subunit α-2	CAPZA2	P47755															↓			
Cofilin-1	CFL1	P23528											↓							
Coronin-1A	CORO1A	P31146								↓										
Coactosin-like protein	COTL1	Q14019					↑													
Dynactin subunit 2	DCTN2	Q13561				↑														
Dynamin-1	DNM1	Q05193		↑ GM, ↓ WM		↑			↑				↑							
Dihydropyrimidi-nase-related protein isoforms	DPYSL2	Q16555	↓	↓ GM and WM	↑	↓	↓	↓	↑	↓		↓	↑	↑				↓		
	DPYSL3	Q14195																		
												
Fascin	FSCN1	Q16658		↓ WM														↓		↑
Glial fibrillary acidic protein	GFAP	P14136	↓				↑			↑			↑	↑		↓		↓	↓	
Gelsolin	GSN	P06396		↓ GM and WM													↑			↓
Α-internexin	INA	Q16352					↑	↑		↓		↓	↑							↓
Microtubule-actin cross-linking factor 1	MACF1	Q96PK2												↑						
Microtubule-asso-ciated protein isoforms	MAP1A	P78559													↑					
	MAP2	P11137													↑					
	MAP6	Q96JE9													↑					
Moesin	MSN	P26038		↑ GM																
Neurofilament intermediate proteins	NEFL	P07196					↓	↓	↓		↓		↓			↓	‒	↓	↓	↓
	NEFM	P07197					↓				‒					↓	↓			↓
	NEFH	P12036									↓									↑
Septin isoforms	SEPT3	Q9UH03		↓ GM and WM					‒	↑	‒								‒	
	SEPT5	Q99719							↑		‒								↓	
	SEPT11	Q9NVA2							↑		↓									
Spectrin α chain, non-erythrocytic 1	SPTAN1	Q13813		↓ GM and WM												↓				
Stathmin	STMN1	P16949				↑	↑				↑						↑	↑		↑
Tubulin isoforms	TUBB	P07437	‒	‒		‒	‒	‒				↑		↓	‒	↑	‒			↑
	TUBA1B	P68363		‒		↓	‒	‒						↓	‒		↓			↓
	TUBB2A	Q6FGZ8		↓ GM and WM			↓	‒						‒	↑		‒			‒
	TUBB3	Q13509						‒						↑			‒			↑
	TUBB4A	P04350						↑									↓			↓
	TUBAL3																			↑
	TUBB2B																			↓
	TUBB4B																			↑
	TUBB6																			↑
Tubulin-folding cofactor B	TBCB	Q99426						↑												
T-complex protein 1 subunit α	TCP1	P17987							↑											
Tropomodulin-2	TMOD2	Q9NZR1								↓										
Tropomyosin isoforms	TPM3	P06753												↓				‒		‒
	TPM4	P67936																↑		↑
Vimentin	VIM	P08670									↑				↑		↑			↑

**Table 2:** Summary of the proteins altered expression from postmortem proteomic investigations of major depressive disorder. Downregulated (↓), upregulated (↑).

			References			
Protein Name	Gene Symbol	Uniprot acc. No.	[26]	[24]	[49]	[41]
Coronin-1A	CORO1A	P31146				↓
Dihydropyrimidinase-related protein isoforms	CRMP1	Q14194	‒	↑	‒	
	DPYSL2	Q16555	↓	↑	↑	
	DPYSL3	Q14195			↑	
Glial fibrillary acidic protein	GFAP	P14136	↓			↓
Neurofilament intermediate proteins	NEFL	P07196				↑
Microtubule-associated protein isoforms	MAP1LC3A	Q9H492			↑	
Tubulin isoforms	TUBA1B	P68363		↑	‒	
	TUBA4B	Q9H853			↑	
Transgelin-3	TAGL3	Q9UI15		↓		
Profilin	PFN1	P07737			↑	
	PFN2	P35080			↑	

**Table 3:** Summary of the proteins altered expression from postmortem proteomic investigations of bipolar disorder. Downregulated (↓), upregulated (↑).

			References					
Protein Name	Gene Symbol	Uniprot acc. No.	[26]	[24]	[31]	[18]	[39,40]	[41]
Actin isoforms	ACTB	P60709					↑	
Actin-related protein 2/3 complex components	ARPC5	O15511					↑	
Dihydropyrimidinase-related protein isoforms	CRMP1	Q14194	‒		‒	‒	‒	
	DPYSL2	Q16555	↓		‒	↑	↓	
	DPYSL3	Q14195			↑	↑	↓	
Dynactin subunit 2	DCTN2	Q13561					↓	
Dynamin-1	DNM1	Q05193					↓	
Fascin	FSCN1	Q16658					↓	
Glial fibrillary acidic protein	GFAP	P14136	↓					
Α-internexin	INA	Q16352			↓		↓	
Kinesin light chain 2	KLC2	Q9H0B6			↑			
Microtubule-associated protein isoforms	MAP1LC3A	Q9H492						
Neurofilament intermediate proteins	NEFL	P07196			‒	‒	↓	↓
	NEFM	P07197			↓	↑	↓	
	NEFH	P12036				↓		
Septin isoforms	SEPT3	Q9UH03	‒		‒		‒	
	SEPT5	Q99719			↑		‒	
	SEPT6	Q14141			↑		‒	
	SEPT11	Q9NVA2			↑		↓	
Spectrin α chain, non-erythrocytic 1	SPTAN1	Q13813					↓	
Stathmin	STMN1	P16949					↑	
Tubulin isoforms	TBBX	P07437		↓	‒			
	TUBA1B	P68363			↑			
	TUBA4B	Q9H853			‒			
	TUBB1	Q9H4B7			↑			
T-complex protein 1 subunit α	TCP1	P17987			↑			
Tropomyosin isoforms	TPM4	P67936					↓	
Vimentin	VIM	P08670				↑	↓	
Programmed cell death 6-interacting protein	PDCD6IP	Q8WUM4				↑		
WD repeat-containing protein 1	WDR1	O75083					↓	

**Table 4:** Proteomic reports in various tissues from schizophrenia, major depressive disorder and bipolar patients. ACC: Anterior cingulate cortex; ATL: Anterior temporal lobe; ATPFC: Anterior prefrontal cortex; CC: Corpus callosum; DLPFC: Dorsolateral prefrontal cortex; FC: Frontal cortex; IC: Insular cortex; ICPL: Isotope-coded protein labelling; SRM: Selected reaction monitoring; WA: Wernicke’s area.

Study (Year)	Region of the Brain Analysed	Technique Used	Ref.
Johnston-Wilson *et al.* (2000)	FC	2-DE	[26]
Prabakaran *et al.* (2004))	DLPFC	2D-DIGE	[27]
Clark *et al.* (2006)	ACC	2-DE	[28]
Beasley *et al.* (2006)	ACC	2-DE	[24]
Sivagnanasundaram *et al.* (2007)	CC	2-DE	[29]
Clark *et al.* (2007)	ACC	2-DE	[30]
Pennington *et al.* (2008)	DLPFC	2-DE/shotgun	[31]
Pennington *et al.* (2008)	IC	2D-DIGE	[32]
English *et al.* (2009)	DLPFC	2D-DIGE	[18]
Behan *et al.* (2009)	DLPFC	2D-DIGE	[33]
Martins-de-Souza *et al.* (2009)	DLPFC	2-DE	[34]
Martins-de-Souza *et al.* (2009)	WA	2-DE	[35]
Martins-de-Souza *et al.* (2009)	DLPFC	Shotgun (ICPL)	[36]
Martins-de-Souza *et al.* (2009)	ATL	Shotgun (ICPL)	[37]
Nesvaderani *et al.* (2009)	Hippocampus	2-DE	[38]
Focking *et al.* (2011)	Hippocampus	2D-DIGE	[39]
Schubert *et al.* (2015)	Hippocampus	2D-DIGE	[40]
Wesseling *et al.* (2014)	APFC	SRM	[41]
Saia-Cereda *et al.* (2015)	CC	Shotgun	[42]
Martins-de-Souza *et al.* (2012)	DLPFC	Shotgun	[49]
